# The complete chloroplast genome sequence of the medicinal plant *Dimetia hedyotidea* (DC.) T.C.Hsu (Rubiaceae) and its phylogenetic analysis

**DOI:** 10.1080/23802359.2026.2694152

**Published:** 2026-06-29

**Authors:** Ade Prasetyo Agung, Ausana Wongtayan, Yihui Tan, Liangqiang Huang, Xinmin Pan, Guoan Shen

**Affiliations:** ^a^ Center for Integrative Conservation & Yunnan Key Laboratory for the Conservation of Tropical Rainforests and Asian Elephants, Xishuangbanna Tropical Botanical Garden, Chinese Academy of Sciences, Mengla, Yunnan, China; ^b^ University of Chinese Academy of Sciences, Beijing, China; ^c^ Department of Pharmacology and Physiology, Faculty of Pharmaceutical Sciences, Chulalongkorn University, Bangkok, Thailand; ^d^ Guangxi Hongyao Biotechnology Co., Ltd., Liuzhou City, China; ^e^ Institute of Medicinal Plant Development, Chinese Academy of Medical Sciences & Peking Union Medical College, Beijing, China

**Keywords:** *Dimetia hedyotidea*, chloroplast, genome, phylogenetic analysis

## Abstract

We assembled and characterized the complete chloroplast genome of *Dimetia hedyotidea* (DC.) T.C.Hsu. It is 154,385 bp with 37.6% GC. In total, 128 genes were annotated, consisting of 83 protein-coding genes, 37 tRNA genes, and eight rRNA genes. The order and composition of genes were similar to those of other Spermacoceae species. A phylogenetic tree based on the chloroplast genome showed that *D. hedyotidea* is nested within Spermacoceae and is sister to *Exallage chrysotricha*. This study enriches the chloroplast genome database for Rubiaceae and provides a foundation for future phylogenetic and genomic studies of the family.

## Introduction

*Dimetia hedyotidea* (DC.) T.C.Hsu, a species within the Rubiaceae family, is predominantly cultivated in the southern regions of China. This plant is well-known for its role in traditional Chinese medicine (TCM), where it is consumed as a sweet herbal tea (Zhao et al. 2021). The tea, made from its leaves, has been used for centuries to treat various ailments, including colds, coughs, and inflammation. Moreover, *D. hedyotidea* has been valued for its antioxidant and anti-inflammatory effects, which contribute to its popularity as a natural remedy (Peng et al. 1998). However, its taxonomic identity has been obscured by frequent synonymization with genera like *Hedyotis* L. and *Oldenlandia* L., creating persistent uncertainty that hampers the precise interpretation of phytochemical and pharmacological studies associated with it.

The seed and pollen morphology of *D. hedyotidea* have been studied, revealing distinct characteristics that contribute to its identification and classification within the genus (Neupane et al. 2015; Hsu and Chen 2017). Although this species was phylogenetically placed within *Dimetia* (Neupane et al. 2015), its formal transfer to the genus was later established by Hsu and Chen (2017). It is imperative to note that since the type specimen was not examined, caution is warranted regarding the species delineation, which may introduce taxonomic uncertainty. However, despite these morphological studies, the phylogenetic position of *D. hedyotidea* remains poorly understood. In this study, the chloroplast genome of *D. hedyotidea* was assembled and annotated. By doing so, we seek to explore its phylogenetic relationships with closely related species, thereby contributing additional molecular evidence to our understanding of the evolutionary history of *D. hedyotidea*.

## Materials and methods

**Figure 1.**
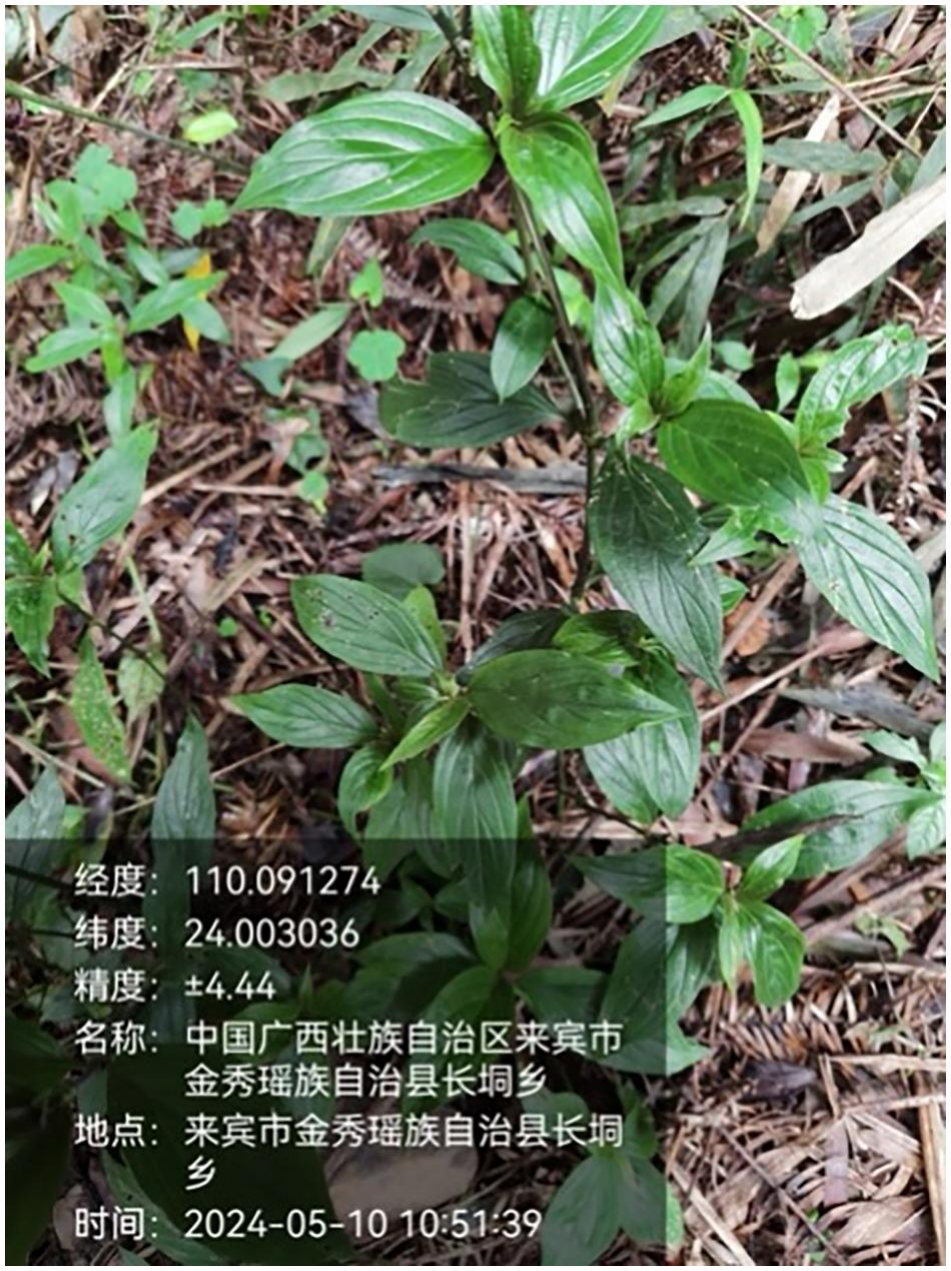
Photograph of *D. hedyotidea.* Photo was taken by Xinmin Pan (one of the authors).

Fresh leaves of one individual (Figure 1) were collected from Changdong Ethnic Township, Jinxiu Yao Autonomous County, Laibin City, Guangxi Zhuang Autonomous Region, China (N 24.003036°, E 110.091274°, 500 m) and preserved in silica gel (Chase and Hills 1991). The specimens were authenticated by Xinmin Pan (40971972@qq.com). The voucher specimen was deposited in the herbarium of the Institute of Medicinal Plant Development (IMPD) under the voucher number Y076 (contact: Guoan Shen, gashen@implad.ac.cn). High-quality genomic DNA was obtained using the DNeasy Plant Mini Kit (Cat. 69104, Qiagen).

**Figure 2.**
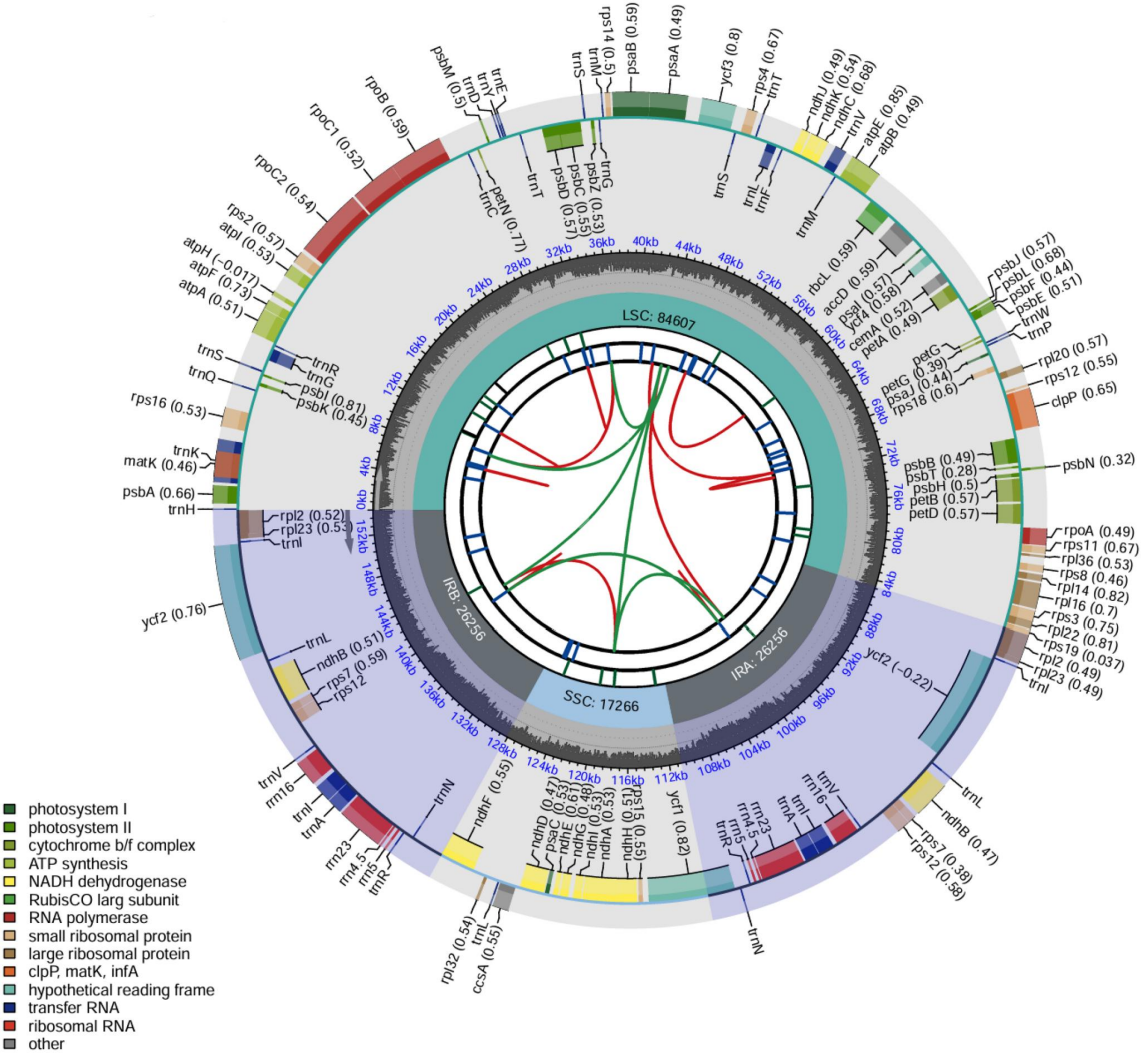
Circular map of *Dimetia* chloroplast genome, generated by CPGview. From the outside inward, the first circle shows transcription directions of gene. The inner and outer genes are clockwise and anticlockwise, respectively. The different functional groups are signed by color-code at the left bottom. The optional codon usage bias is displayed next to the gene name. The number in outer of second circle is position in chloroplast genomes. The dark gray parts in the inner second circle shows GC content with cutoff 50% (dotted line). The third circle represents four parts of the chloroplast genome, consisting of LSC (large single-copy, green part), SSC (small single-copy, blue part), IRA and IRB (inverted repeat regions, gray part). The fourth circle shows the long repeats whose location are marked by blue bars. The fifth circle describes microsatellite sequences as short bars with different colors. The colors represent the type of repeat. Further details are provided in the CPGview homepage.

A DNA library with an insert size of approximately 300 base pairs (bp) was constructed, followed by paired-end sequencing (2 × 150 bp) using the Illumina HiSeq 2500 platform. Approximately 14.7 Gb of raw data from *D. hedyotidea* were obtained. Quality control of the raw sequencing reads was performed with fastp (v1.3.1), producing high-quality filtered reads. These reads were subsequently used for the de novo assembly of complete chloroplast genomes utilizing the GetOrganelle toolkit (v.1.7.7.0; Jin et al. 2020) with the parameters ‘-R 15 -k 21,45,65,85,105 -F embplant_pt’. To confirm the accuracy of the assembly, the chloroplast genome was visualized using Bandage v.0.8.1 (Wick et al. 2015). Annotations were completed using a combination of GeSeq (Tillich et al. 2017) and CPGAVAS2 (Shi et al. 2019). The coverage depth ranged from a minimum of 2,123× to a maximum of 10,066× (Figure S1). All gene features, including cis-splicing (Figure S2), trans-splicing (Figure S3), and the circular map (Figure 2), were visualized using CPGView (Liu et al. 2023).

We created a dataset consisting of this newly assembled chloroplast genome and 13 other chloroplast genomes retrieved from NCBI (Table S1). The 13 chloroplast genomes were selected to represent four tribes within Rubioideae (Dunnieae, Foonchewieae, Rubieae, and Spermacoceae) based on the availability of high-quality, complete chloroplast genomes in NCBI. This sampling strategy prioritizes taxonomic breadth and alignment reliability over exhaustive inclusion of all available Spermacoceae sequences. Additionally, *Dunnia sinensis* (MN883829.1) and *Foonchewia coriacea* (MT942688.1) were chosen as the outgroup. All protein-coding gene (PCG) regions from each chloroplast genome were extracted following the script of ‘get_annotated_regions_from_gb.py’ (https://github.com/Kinggerm/PersonalUtilities/). The extracted CDS genes were aligned using MACSE v2 (Ranwez et al. 2018), trimmed by removing gaps using trimAl v.1.2 (Capella-Gutierrez et al. 2009), and concatenated using PhyloSuite v1.2.3 (Zhang et al. 2020).

The maximum-likelihood phylogeny was reconstructed using the IQ-TREE web server (Trifinopoulos et al. 2016) by running 1,000 replicates and 1,000 UFBoot (UltraFast BootStraps) (Hoang et al. 2018), with Q.BIRD+F + I + G4 selected as the best-fit model according to Bayesian Information Criterion (BIC).

## Results

The chloroplast genome exhibited a length of 154,385 bp and had a typical quadripartite structure, consisting of a large single copy (84,607 bp), a small single-copy region (17,266 bp), and two inverted repeat regions (26,256 bp each). The GC content was 37.6%.

**Figure 3.**
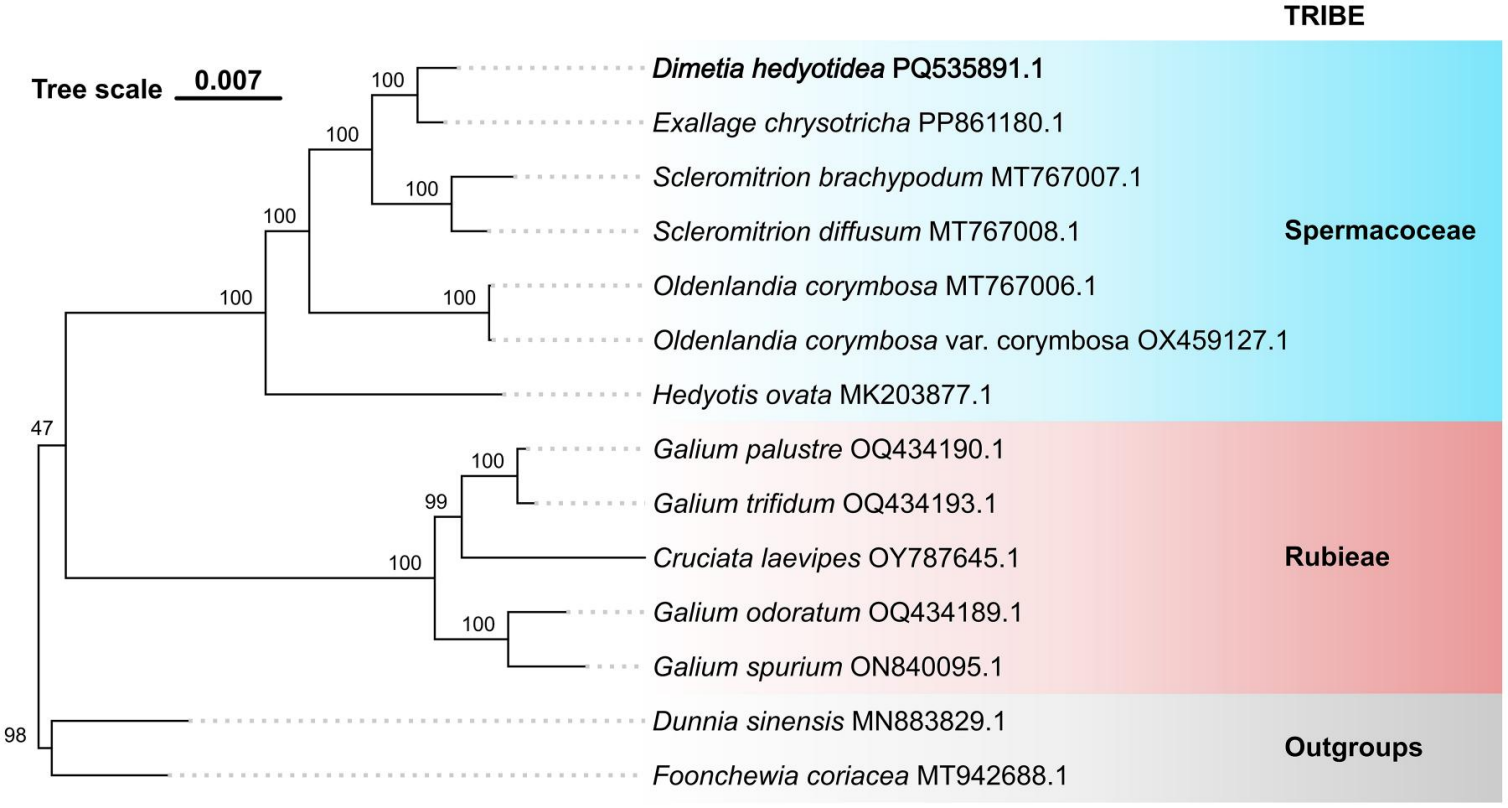
A Maximum-likelihood tree showing that the studied species, *Dimetia hedyotidea*, PQ535891.1 (bold), is monophyletic with other species from the Spermacoceae tribe, including *Hedyotis ovata* (MK203877.1, Zhang et al. 2019); *Scleromitrion brachypodum* (MT767007.1, Yik et al. 2021); *Scleromitrion diffusum* (MT767008.1, Yik et al. 2021), *Exallage chrysotricha* (PP861180.1); and *Oldenlandia corymbosa* (MT767006.1, Yik et al. 2021 and OX459127.1). Additional tribes include Rubieae (*Galium spurium*, ON840095.1, Yin et al. 2023; *Galium odoratum*, OQ434189.1, *G. palustre*, OQ434190.1, *G. trifidum*, OQ434193.1, Ciborowski et al. 2024; *Cruciata laevipes*, OY787645.1). Dunnieae (*Dunnia sinensis*, MN883829.1, Zhang et al. 2020); Foonchewieae (*Foonchewia coriacea*, MT942688.1, Zhang et al. 2021) were used as the outgroup. Number on the nodes indicate SH-aLRT/UFBS values.

A total of 128 functional genes were annotated, among which 83 are protein-coding genes (PCGs), 37 are transfer RNA genes (tRNAs), and eight are ribosomal RNA genes (rRNAs). Within the IR regions, there are duplications of seven PCGs (*ycf1*, *rps12*, *rps7*, *ndhB*, *ycf2*, *rpl23*, and *rpl2*), seven tRNAs (*trnN-GUU*, *trnR-ACG*, *trnA-UGC*, *trnI-GAU*, *trnV-GAC*, *trnL-CAA*, and *trnI-CAU*), and four rRNAs (*rrn5*, *rrn4.5*, *rrn23*, and *rrn16*). Furthermore, there are ten PCGs that contain a single intron (*rps16*, *rpoC1*, *petB*, *petD*, *rpl16*, *rpl2* (2×), *ndhB* (2×)), and *ndhA*), and two genes (*ycf3* and *clpP*) contain two introns (Figure S2). Meanwhile, there are eight tRNA genes that contain a single intron (*trnK-UUU*, *trnS-CGA*, *trnL-UAA*, *trnV-UAC*, *trnI-GAU* (2×), and *trnA-UGC* (2×). The *rps12* gene undergoes trans-splicing, which separates it into three unique exons, two of which are duplicated in the IR regions (Figure S3). The phylogenetic relationship analysis showed that *D. hedyotidea* is sister to *Exallage chrysotricha* with highly supported values (Figure 3).

## Discussion and conclusion

The species *Dimetia hedyotidea* belongs to the *Oldenlandia-Hedyotis* complex (Neupane et al. 2015). It has been placed into different genera, such as *Exallage*, *Hedyotis*, and *Oldenlandia*, when this complex taxon is discussed. However, in this study we used the genus name *Dimetia* to further discuss our findings (WFO 2025). The complete chloroplast genome of *D. hedyotidea* was successfully assembled and characterized in this study. The genome size was determined to be 154,385 bp. This size falls within the range of other Spermacoceae species, from 152,327 (*Oldenlandia corymbosa,* MT767006.1) to 154,560 bp (*Hedyotis ovata*, MK203877.1).

The conserved chloroplast genome structure and gene content of *D. hedyotidea*, similar to those of other Spermacoceae species, suggest that chloroplast genome evolution in this group has been relatively stable. The phylogenetic placement of *D. hedyotidea* as sister to *E. chrysotricha*, based on the concatenated protein-coding genes, provides additional molecular evidence supporting the generic realignment proposed by Hsu and Chen (2017).

Nevertheless, we caution that phylogenies based exclusively on chloroplast genomes may not fully resolve complex taxonomic relationships, especially in groups with possible hybridization or incomplete lineage sorting. Future studies incorporating nuclear markers are needed to further clarify the taxonomic status of *D. hedyotidea*.

## Supplementary Material

Supplemental Material

Supplemental Material

Supplemental Material

## Data Availability

The genome sequence data that support the findings of this research are available in GenBank under the accession number PQ535891. The BioProject, BioSample, and SRA numbers are PRJNA1170469, SAMN44107158, SRR30920788, respectively.
