# Supplementary figures and images for: The complete chloroplast genome sequence of the medicinal plant Dimetia hedyotidea (DC.) T.C.Hsu (Rubiaceae) and its phylogenetic analysis
Source: Mitochondrial DNA B Resour. 2026 Jun 29;11(8):893–7. doi: 10.1080/23802359.2026.2694152 (PMC13410546; doi:10.1080/23802359.2026.2694152)

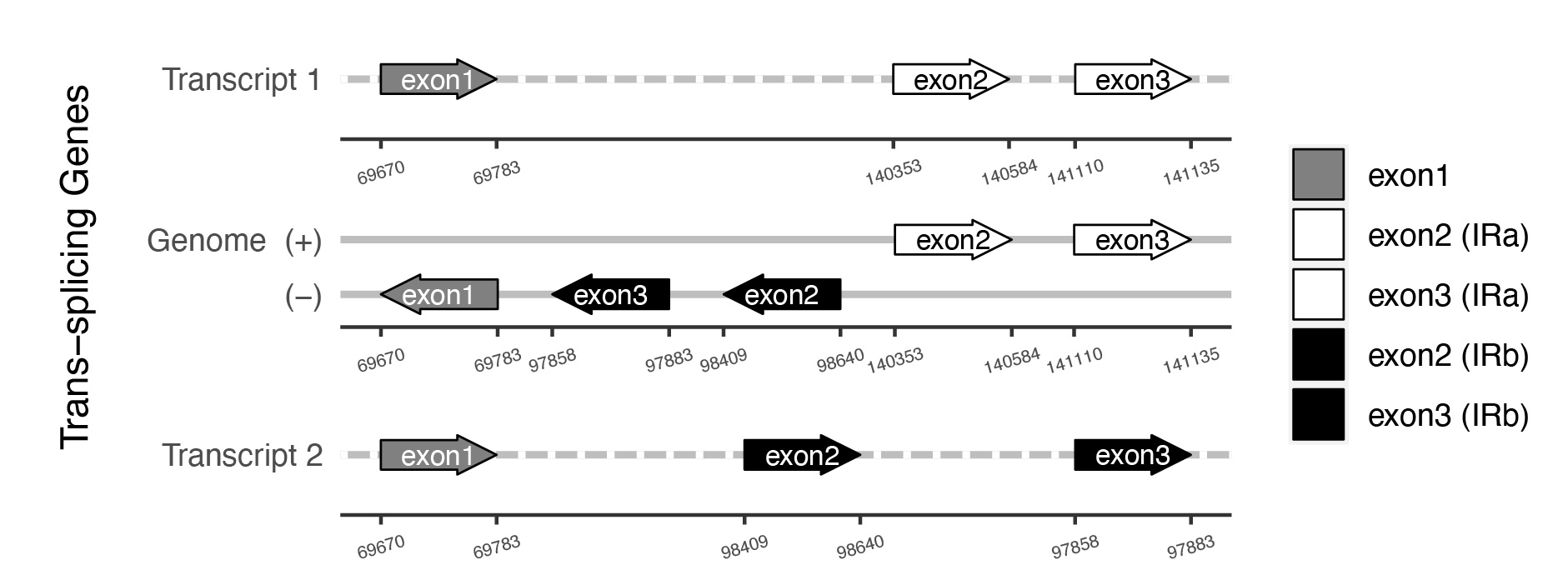

Supplement: Supplemental Material [file TMDN_A_2694152_SM4654.jpg]

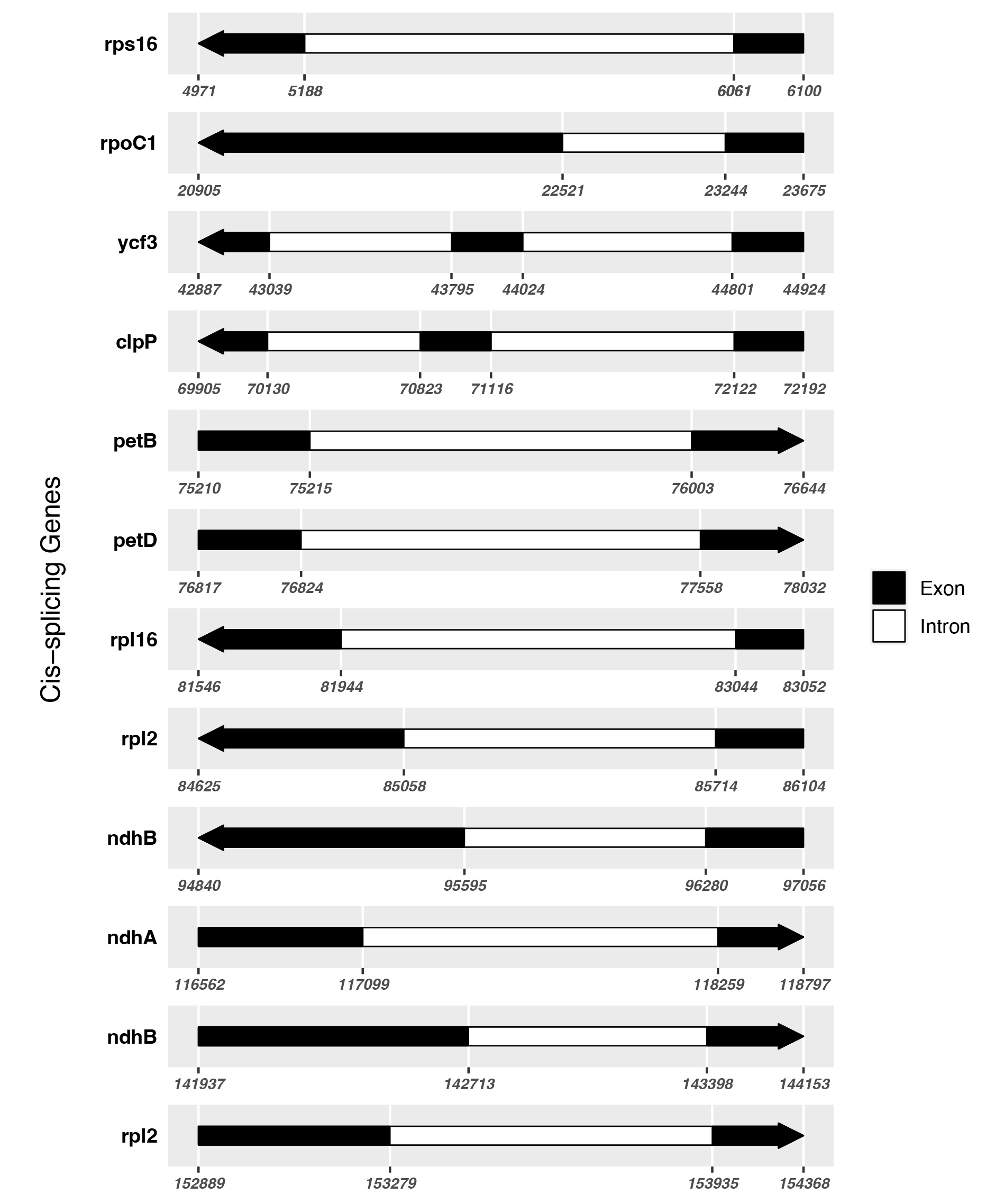

Supplement: Supplemental Material [file TMDN_A_2694152_SM4653.jpg]

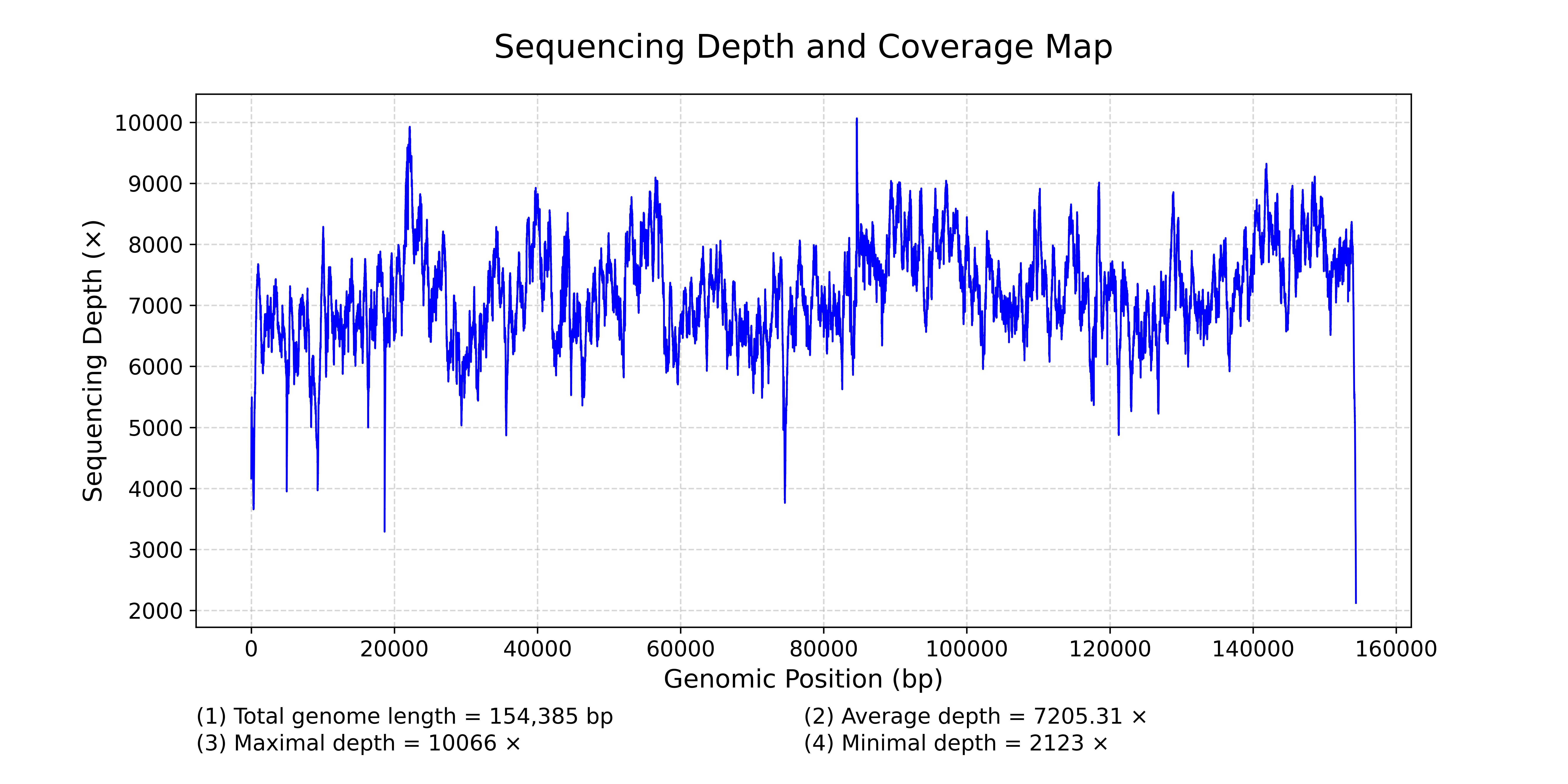

Supplement: Supplemental Material [file TMDN_A_2694152_SM4650.jpg]
